# Stabilization of swine faecal samples influences taxonomic and functional results in microbiome analyses

**DOI:** 10.1016/j.mex.2022.101716

**Published:** 2022-04-29

**Authors:** Xavier C. Monger, Linda Saucier, Alex-An Gilbert, Antony T. Vincent

**Affiliations:** a Département des sciences animales, Faculté des sciences de l'agriculture et de l'alimentation, Université Laval, Quebec City, QC G1V 0A6, Canada; b Institut sur la nutrition et les aliments fonctionnels, Faculté des sciences de l'agriculture et de l'alimentation, Université Laval, Quebec City, QC G1V 0A6, Canada

**Keywords:** DNA preservation, DNA stabilization, Faeces sampling, Microbiome, Shotgun sequencing, Swine

## Abstract

Studies on the microbiome of different species are on the rise, due to a growing interest in animal health and the safety of food products of animal origin. A challenge with studying animals’ microbiomes is to find methods that obtain a good representation of the microbial community of interest. Good unbiased sampling protocols are the basis for a solid experimental design, but may need to be done in environments where sample preservation could be difficult. In this study, we evaluate by shotgun sequencing the impact of stabilizing swine faeces samples using a commercial stabilizer (PERFORMAbiome • GUT | PB-200, DNA Genotek). Using stabilizer makes it possible to obtain DNA that is significantly less degraded than when the samples are not stabilized. Also, the results on the taxonomy and on the bacterial functions encoded in the microbiome are impacted by whether or not the samples are stabilized. Finally, the stabilization of samples that had already been frozen and stored at -80°C led to extraction and DNA quality results similar to those obtained from samples that were stabilized before freezing.

## Highlights

• Stabilization of faecal samples improves the integrity of extracted DNA.
• Microbiome results are affected by sample stabilization.
• Results are similar for samples that were stabilized when frozen, to samples that were stabilized before freezing.

## Graphical abstract

**Figure:**
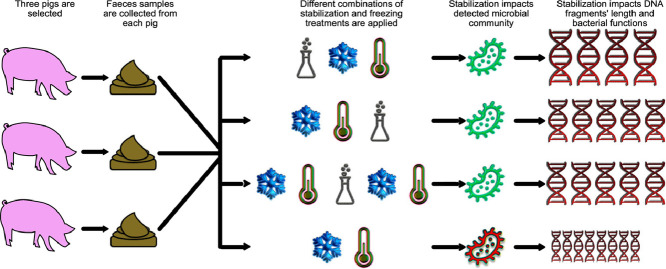

Specifications table

**Table:** 

Subject Area:	Agricultural and Biological Sciences
More specific subject area:	Microbiome
Method name:	Stabilization of swine faecal samples
Name and reference of original method:	Collection and stabilization of DNA using PERFORMAbiome • GUT | PB-200 (DNA Genotek, Ottawa, ON).
Resource availability:	The whole genome shotgun sequencing data set was deposited in the NCBI's Sequence Read Archive (SRA) database under the accession ID PRJNA801645.

## Introduction

The study of the microbiome, defined as the microbial population and its by-products [1], has gained interest in the past decade. The microbiome and health have been linked with a wide variety of diseases, both in animals and humans [2], [3], [4], [5], [6]. The microbiome is known to play a decisive role at the metabolic level, for example in the production of short chain fatty acids [7], as well as in various immune functions [8] and in the protection against pathogens such as *Clostridium difficile, Salmonella* and other pathogenic enterobacteria [6]. In swine, the microbiome is known to be involved in porcine reproductive and respiratory syndrome [9] and chronic kidney disease [10]. The study of microbiomes by molecular techniques has allowed an overview that was not possible with culture-based methods. However, biases such as over- or under-representation of taxa or functions can easily be introduced at each step of microbiome studies, from sample collecting to metagenomic analyses [11].

The standardization of sample collection is highly challenging, as samples may be heterogeneous, collected from many sources and in remote environments (where appropriate cold or freezing storage may not be readily available). Without stabilization, the sample's microbial community is likely to change during transit, and their molecular components may deteriorate before the DNA is extracted [12], [13], [14], therefore influencing the metagenomic results obtained. To overcome this problem, commercial stabilizers have been developed to preserve the microbial community and its DNA while removing the need for immediately freezing the samples [15], [16], [17]. Stabilizers can be employed for sampling in remote field locations, for the delivery of biological samples, or for at-home sampling kits for humans. However, their efficacy and their impacts on the metagenomic results have not yet been extensively nor independently studied and remain largely as trade knowledge.

In this study, we evaluated the impact of DNA stabilization, not only upon collection, but also during thawing of previously frozen samples, on metagenomic results. DNA samples from pig faeces treated at various steps with a commercial stabilizer (PERFORMAbiome • GUT | PB-200, DNA Genotek, Ottawa, ON) were sequenced by a shotgun approach at great depth and analyzed. We were able to verify the influence of stabilization in three different ways: (1) the quality of the extracted DNA, (2) the taxonomic assignment of the bacterial population, and (3) the bacterial functions encoded by the microbiome.

## Methods

### Animal housing and treatment conditions

The samples analyzed were a subset from a larger experiment whose aim is to study the impact of different supplements (an antibiotic and a probiotic) on the intestinal microbiota of pigs. The animals were treated as required by the guidelines of the National Farm Animal Care Council [18]. The experimental design was also approved by the Animal Protection Committee of Université Laval prior to the experiments (2019057-1). Six Yorkshire-Landrace male pigs (25 kg) were obtained from the same reproduction nursery farm and were assigned two per experimental group, according to a cross-over design. Upon castration at their farm of origin, they received an antibiotic mix consisting of trimethoprim and sulfadoxine; they were suckling piglets at the time of this procedure. At weaning, the pigs were vaccinated with Ingelvac CircoFLEX vaccine (Boehringer Ingelheim, Burlington, ON, Canada). When the pigs reached the age of 55 days, they were transported to the animal facility at Université Laval. The pigs were then given an 11-day period to adapt to their new environment, before proceeding to an ileum canulation surgery. Prior to the procedure, the pigs were fasted for 16 h. The surgery, along with preparation and post-procedure care, was performed as described by Wubben et al. [19]. The pigs were returned to their full feeding rations 24 h after surgery and were allowed 11 days to recover before the start of the treatment phase. During adaptation and recovery from the surgery, animals received a commercial control diet. This surgery was necessary to collect digestate samples for the main experiment, which is not described in the present study, which focuses on the impact of stabilization of pig faeces.

**Fig. 1:**
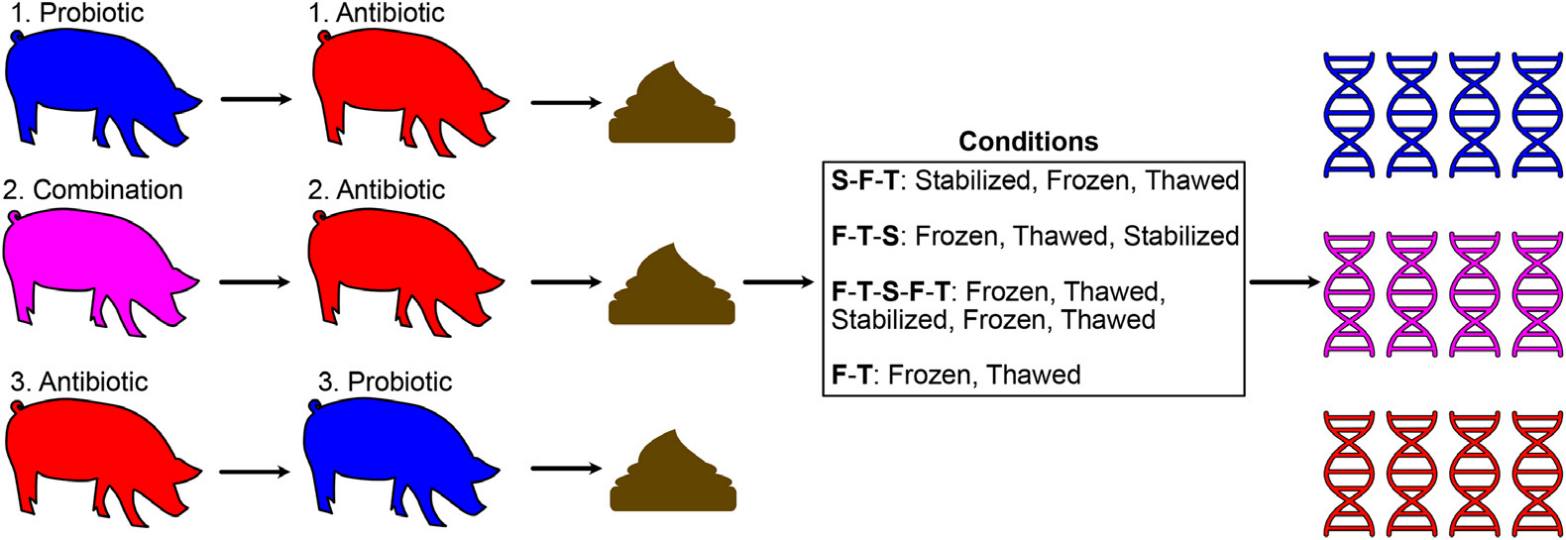
Subset of samples taken randomly from a larger pig experiment. Schematic representation of the treatments applied to the pigs from which the faeces were collected.

The subset of samples was obtained from pigs treated as shown in Fig. 1. The probiotic treatment consisted of *Pediococcus acidilactici* MA18/5M (PA; 10^8^ CFUs/day; Biopower^Ⓡ^ PA, Lallemand Animal Nutrition, Milwaukee, WI, USA), the antibiotic treatment consisted of tylvalosin (TYL; 250 g/ton of feed; Aivlosin^Ⓡ^ (17% Tylvalosin), Eco Animal Health, Princeton, NJ, USA). The combination treatment was composed of the antibiotics and probiotics administered simultaneously. The probiotic treatment was administered by feeding the pigs with a small commercial egg-free pie dough ball (3 g) containing the lyophilised *P. acidilactici* cells covered with a drop of molasses; this treatment was administered daily. The pig feed used for the experiment was a commercial finishing feed (Sollio Agriculture, St-Romuald, QC, Canada) composed of corn, wheat, soy-based ingredients, and micronutrients.

### Faeces sampling and DNA extraction

Faeces were aseptically collected and either stabilized, or not, in PERFORMAbiome • GUT | PB-200 tubes according to the manufacturer's instructions. Both samples were collected and transported on ice before being frozen at –80°C upon return to the laboratory.

Four conditions were tested on the faeces collected (Fig. 1). The first, S-F-T, corresponds to faeces stabilized (S) on-farm, frozen (F) and thawed (T). The second, F-T-S, corresponds to faeces frozen, thawed at 4°C for 7 h, and stabilized. The third is like the second, except that there is an additional freeze/thaw cycle after stabilizing the faeces (F-T-S-F-T). The fourth condition is simply frozen and thawed faeces without stabilization (F-T). While the S-F-T and F-T treatments correspond to the conditions expected in the field (directly stabilized or not stabilized), the F-T-S and F-T-S-F-T treatments make it possible to verify whether stabilization impacts already frozen samples and whether it offers protection against freezing/thawing cycles. Samples were taken from the same faeces for each pig to minimize variability and bias.

Extraction was performed using the QIAamp PowerFecal Pro DNA Kit (QIAGEN, Toronto, Canada) using 250 µL of stabilized faeces or 250 mg of unstabilized faeces. The extracted DNA was eluted with 75 µL of buffer, quantified with the DNA quantification kit PicoGreen (Invitrogen, Waltham, Massachusetts, USA), and preserved at –20°C. DNA integrity was verified using an automated electrophoresis apparatus (Agilent TapeStation System, Agilent Technologies Canada Inc, Mississauga, Canada) with the Genomic DNA ScreenTape Analysis kit (Agilent Technologies Canada Inc).

### Sequencing and metagenomic analysis

The library preparation and sequencing on an Illumina NovaSeq 6000 device were done by the Genome Quebec Centre of Expertise and Services (Montréal, Canada). The reads were first cleaned to remove optical duplicates with the clumpify tool from BBTools version 38.92 [20]. The reads were then filtered using Trimmomatic version 0.39 [21] and mapped on the *Sus scrofa* reference genome Sscrofa11.1 (RefSeq: GCF_000003025.6) using a combination of Bowtie version 2.4.4 [22] and SAMtools version 1.13 [23] to remove reads from the pig host. The number of reads kept at each step is shown in Table 1. Finally, the cleaned reads were analysed using the SqueezeMeta pipeline version 1.4.0 with the default parameters and the coassembly option [24]. The package SQMtools [25] was used to integrate SqueezeMeta data in R and for the functional analysis. Taxonomic and functional values were converted to relative abundances to compare results between samples. Vegan [26] was used to assess the alpha diversity of the samples. Phyloseq [27] and Microbial [28] were used to analyse the taxonomy and beta diversity. The whole genome shotgun sequencing data set was deposited in the NCBI's Sequence Read Archive (SRA) database under the accession ID PRJNA801645 (Table 1).

**Table 1:** Number of millions of paired reads kept at each cleaning step.

Sample	Initial	After deduplication	After filtration	After host removal	Accession
S-F-T1	105.20	97.57	86.77	86.50	SAMN25371582
S-F-T2	96.92	90.31	84.48	84.39	SAMN25371583
S-F-T3	108.77	100.90	91.11	90.96	SAMN25371584
F-T-S1	112.24	104.37	93.31	93.17	SAMN25371576
F-T-S2	104.79	97.33	91.46	91.33	SAMN25371577
F-T-S3	107.68	99.75	93.35	93.25	SAMN25371578
F-T-S-F-T1	91.42	85.05	76.99	76.84	SAMN25371579
F-T-S-F-T2	107.59	99.73	93.25	93.10	SAMN25371580
F-T-S-F-T3	99.25	91.78	84.66	84.55	SAMN25371581
F-T1	69.21	61.19	51.18	51.00	SAMN25371574
F-T2	113.52	100.00	89.58	88.97	SAMN25371573
F-T3	80.95	75.38	69.31	68.99	SAMN25371575

### Statistical analysis

The values of DNA integrity were analysed with Prism version 9.2.0 using a Kruskal-Wallis multiple comparison with a Dunn's correction. The alpha diversity of samples was analysed using the same tests. The statistical analysis of the function markers were done with the ldamarker function of Microbial package [28].

## Method validation

### Impact of stabilization treatments on the integrity of DNA

**Fig. 2:**
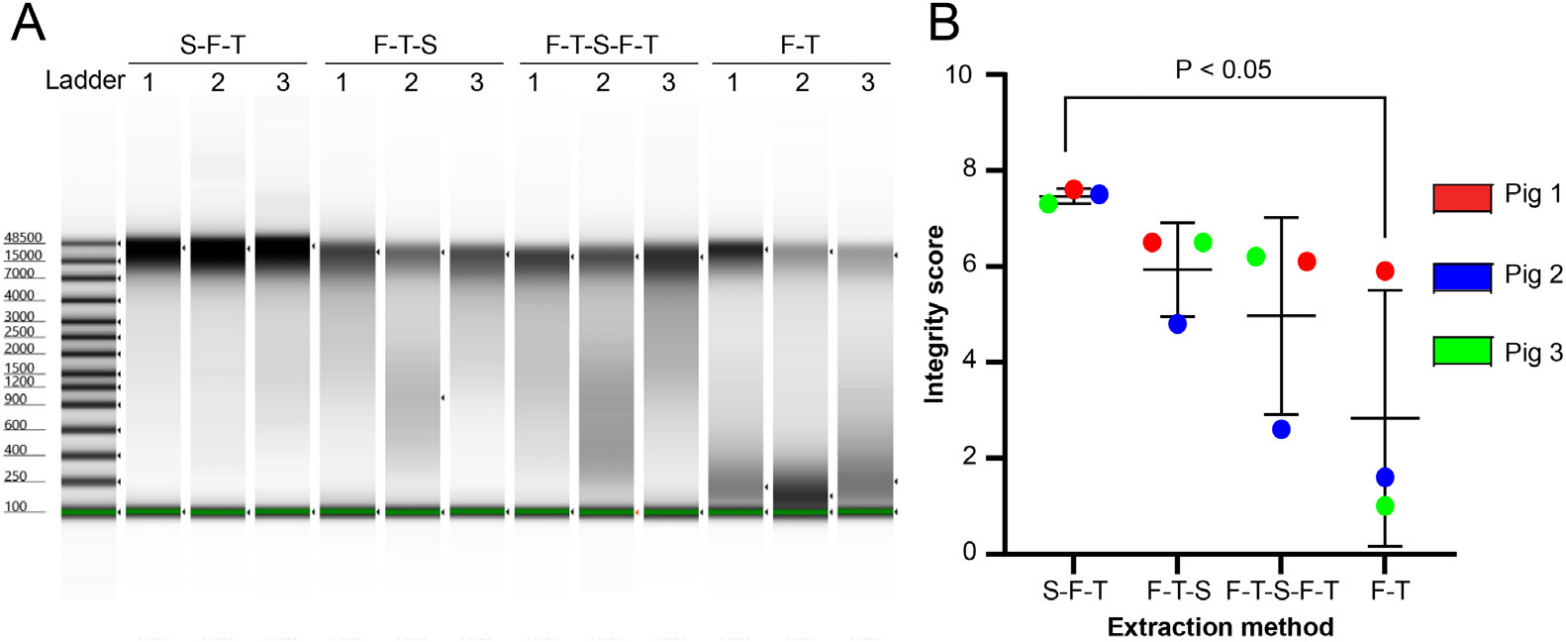
Assessment of DNA quality according to treatments. (A) Computer generated gel of the DNA extracted from faecal samples and (B) DNA Integrity Number (DIN) for all methods of stabilization. S-F-T = stabilized, frozen, thawed; F-T-S = frozen, thawed, stabilized; F-T-S-F-T = frozen, thawed, stabilized, frozen, thawed; F-T = frozen, thawed.

Visually, DNA extracted from unstabilized samples (F-T) were more degraded than DNA from samples stabilized at the farm (S-F-T), based on the smear present on a gel-like electropherogram (Fig. 2A). The DNA Integrity Numbers (DIN), generated by the TapeStation System, for the S-F-T samples are significantly higher than for the unstabilized samples (F-T; Fig. 2B). Furthermore, the standard deviation was less for the DIN of DNA from S-F-T samples compared to the other treatments. The integrity of DNA samples stabilized after freezing (F-T-S and F-T-S-F-T) were found to be intermediate between S-F-T and F-T samples and were not statistically different with any other treatments.

### Impact of the stabilization treatment on the microbial community

**Fig. 3:**
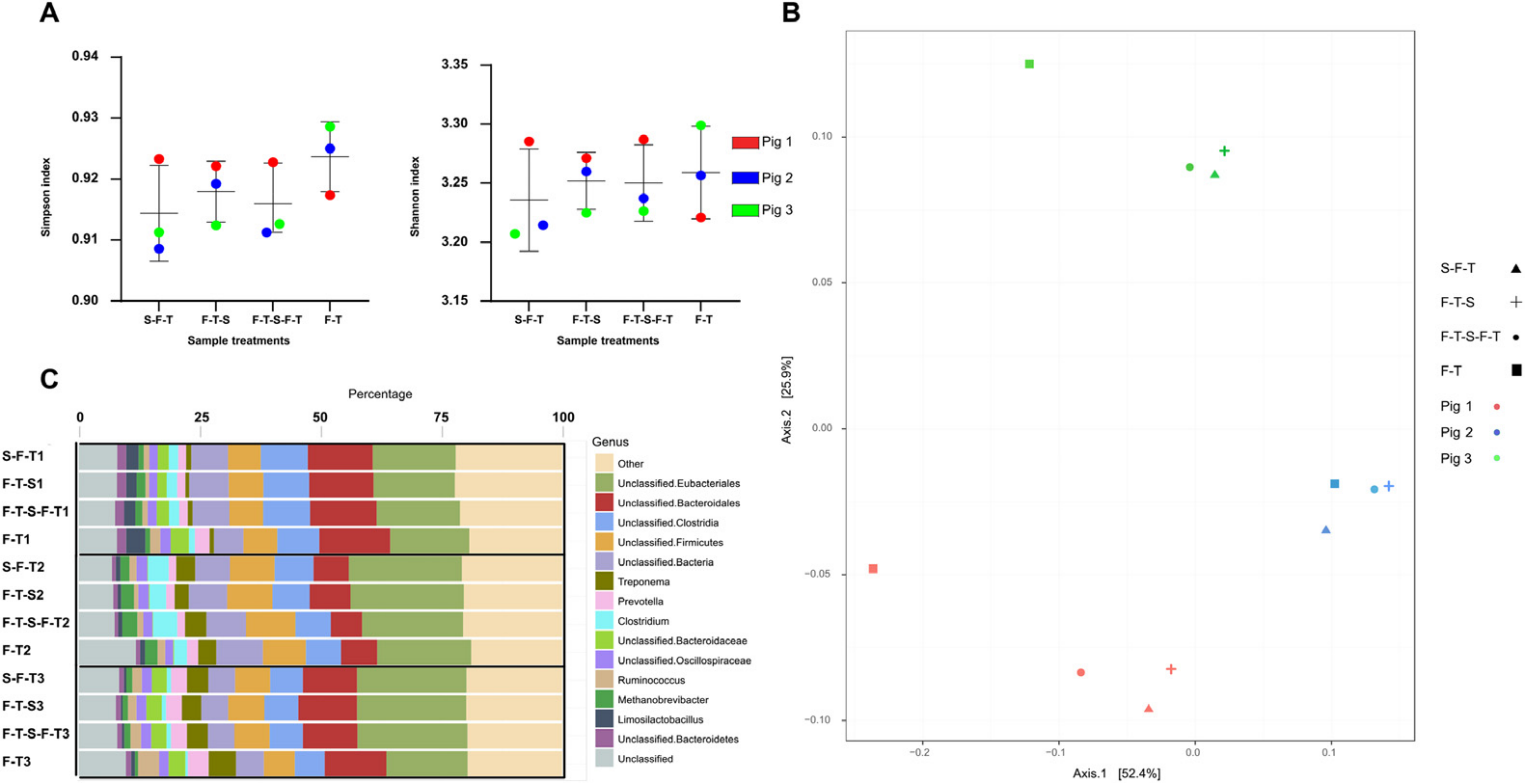
Effect of the treatments on the taxonomic study of the samples. Plots of the (A) alpha diversity (Simpson and Shannon indexes) of the samples (B) beta diversity of the samples (PCoA) based on Bray-Curtis dissimilarity, and (C) taxonomic classification of the main taxa identified in the faecal samples with different stabilizations. S-F-T = stabilized, frozen, thawed; F-T-S = frozen, thawed, stabilized; F-T-S-F-T = frozen, thawed, stabilized, frozen, thawed; F-T = frozen, thawed.

An analysis of alpha diversity (Simpson and Shannon indexes) shows that whether the samples are stabilized or not does not significantly impact the microbial diversity (Fig. 3A). Principal coordinate analysis (PCoA) of the beta diversity indicates that the samples cluster by pig and not according to the stabilization treatments (Fig. 3B). The F-T sample, however, is outside the cluster of sample treatments from Pigs 1 and 3, but not from Pig 2. This result might be linked with DNA integrity. Compared to Pigs 1 and 3, the DINs of Pig 2 are low for the F-T-S and F-T-S-F-T treatments (Fig. 2). It is therefore possible that the difference between F-T-S, F-T-S-F-T and the F-T for Pig 2 is small compared to the other pigs, thus reflecting the clustering in the PCoA of the beta diversity. The taxonomic composition was highly similar between samples (Fig. 3C), with *Eubacteriales, Bacteroidales, Clostridia* and, *Firmicutes* being the main taxa. The taxonomic composition for F-T samples seems to have more “unclassified” reads compared to the other samples, although not significant (*p* = 0.14). Also, no taxonomic marker specific for any treatment group or taxa were found based on a LEfSe (Linear discriminant analysis Effect Size) analysis.

### Impact of stabilization on functional analysis

**Fig. 4:**
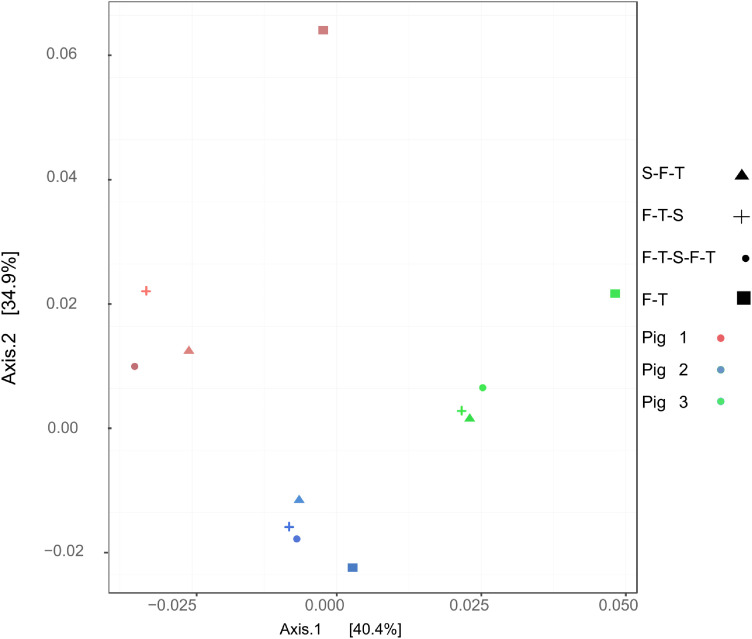
Influence of treatments on predicted metabolic functions in samples. The PCoA is based on the relative abundance and on Bray-Curtis dissimilarity of the different Pfam categories in all the faecal samples for all methods of stabilization and pigs. S-F-T = stabilized, frozen, thawed; F-T-S = frozen, thawed, stabilized; F-T-S-F-T = frozen, thawed, stabilized, frozen, thawed; F-T = frozen, thawed.

The impact of the presence or absence of sample stabilization on the interpretation of the functional diversity results was verified by comparing the identified functions using the Pfam categories, which allows us to classify protein sequences into families and domains [29]. A PCoA of the Pfam categories across samples showed that the clustering by pig was still visible (Fig. 4), similar to what was observed for beta diversity (Fig. 3B). The samples from Pigs 1 and 3 for the F-T treatment clearly do not cluster with the other samples. Again, although the Pig 2 samples cluster together, the sample from the F-T treatment is a little more off-center than those from the other treatments. As for the beta diversity, this result appears to be consistent with DNA integrity, where the difference observed between the samples may be small given the high level of Pig 2’s sample degradation (Fig. 2).

The bacterial functions were significantly different between the S-F-T or F-T samples (Table 2). Only samples from these treatments were considered for marker identification since they show the most contrasting results regarding the level of degradation and reflect the two protocols most likely to be used for metagenomics projects: a recommended use of stabilizer (directly at the farm) and collection without stabilization. While no taxa were statistically different between these treatments based on a LEfSe analysis, 10 functions were found to be significantly different and by at least twofold (Log2 > 1 or < -1). Only two of them were found more frequently in the S-F-T samples, while the other eight functions were amplified in the F-T samples. Some of the categories found more frequently in the F-T samples are involved in mobile DNA elements, such as phages (PF06841, PF05894) and plasmids (PF01815). It is interesting to note that category PF14581, related to the SseB protein and enriched in F-T samples, is exclusively found in enterobacteria and was experimentally shown to be part of the translocon components of the type III secretion system encoded by the *Salmonella* pathogenicity island 2 in *Salmonella enterica* serovar Typhimurium [30].

**Table 2:** Molecular functions significantly different between S-F-T and F-T treated samples.

Pfam ID	Function description	Log_2_ fold change^a^	P value
PF07065	D123	-1.66	p < 0.01
PF14385	Domain of unknown function	-1.46	p < 0.01
PF14581	SseB protein C-terminal domain	1.01	p < 0.01
PF00942	Cellulose binding domain	1.08	p < 0.01
PF16231	Domain of unknown function	1.14	p < 0.01
PF06841	T4-like virus tail tube protein gp19	1.40	p < 0.01
PF05894	Podovirus DNA encapsidation protein (Gp16)	1.56	p < 0.01
PF01804	Penicillin amidase	1.92	p < 0.01
PF13322	Domain of unknown function	2.45	p < 0.01
PF01815	Rop protein	3.50	p < 0.05

a Negative values represent functions that were overrepresented in the S-F-T group while positive values were overrepresented in F-T group.

Biological sample collection is the essential first step of any microbiome study. It is crucial to optimize sample collection to minimize bias and have a precise idea of the microbial community and its by-products. The results of this study demonstrate that the DNA quality of samples stabilized promptly after collection, at the farm, was significantly higher than unstabilized samples (Fig. 2). This was also observed for other DNA Genotek products made to stabilize oral fluids [31]. Cardona et al. [12] also observed that the absence of stabilization negatively impacts DNA integrity. It is interesting to note that stabilizing already frozen samples (F-T-S and F-T-S-F-T) produces DNA integrity intermediate between S-F-T and F-T treatments. Based on these results, it might therefore be of interest to stabilize already frozen samples, especially for long-read sequencing, for which DNA quality is critical [32].

According to alpha diversities, there was no major difference in microbial abundance between the samples and the treatments (Fig. 3A). Mathay et al. [33] also found no significant difference in alpha diversity between stabilized and unstabilized samples when using the P-084 and P-085 products of DNA Genotek [33]. However, as reported by Anderson et al. with larger sample size, a small increase in alpha diversity was found for stabilized samples with OMNIgene Gut [16], a similar product to PERFORMAbiome Gut studied here. These inconsistencies between studies may arise from the difference between the subjects of the study and the methods used. For example, Anderson et al. [16] worked on human faecal samples, while the present study investigated pig samples. Anderson et al. [16] also used shotgun sequencing, like the present study, however, in our experiment, the samples were sequenced at a greater depth (∼ 80M paired reads versus ∼ 7M paired reads). It is therefore possible that several low proportion taxa are more represented in the present study than for Anderson et al. [16]. Mathay et al. [33] worked on the human faeces microbiome, like Anderson et al. [16], but used 16S amplicon sequencing.

The composition of the most represented genera is also stable (Fig. 3C) between samples and treatments, although unstabilized samples appear to have a slight increase in unclassified taxa. These results are in accordance with Tedjo et al. [34], who demonstrated that the microbial community is generally not affected when faeces are left unstabilized for 24 h when stored at 4°C. One explanation could be that the differences due to the stabilization protocol were smaller than the differences between individuals. This correlates well with the findings of Yeoh et al. who demonstrated that the differences between unstabilized and stabilized samples were highly dependent on the individual providing the samples [13]. Also, the fact that all three pigs received a different treatment could contribute to the fact that the difference due to the pigs is greater than the difference due to the stabilization protocol, although stabilization visibly reduces variability caused by degradation of the samples.

A PCoA of the beta diversity values shows that the unstabilized samples are distant from other sample treatments for the same pigs. This result suggests that although there is no specific effect on one or more bacterial genera, stabilization has an overall effect on the bacterial composition. However, for Pig 2, the samples of the four treatments are more tightly grouped together (Fig. 3B). This result is interesting since it seems to be linked with their DNA integrity (Fig. 2). Compared to Pigs 1 and 3, where only the DNA of the F-T sample is degraded, the DNA of the F-T-S and F-T-S-F-T samples for Pig 2 are also particularly degraded. It is therefore possible that the microbial composition is impacted by the quality of the DNA and that the taxonomic differences between the F-T-S, F-T-S-F-T and F-T samples from Pig 2 are low, because of the high degradation level of these samples.

The fact that, for each of the three pigs, the samples from the F-T-S and F-T-S-F-T treatment clusters with the S-F-T sample in the PCoA of beta diversity shows that stabilization of an already frozen sample achieves results similar to those obtained from directly stabilized samples. This affirmation is corroborated by another study that reported that samples stabilized in PERFORMAbiome Gut collecting tubes were more resistant to freeze/thaw cycles [17]. Interestingly, Wegl et al. demonstrated that stabilization methods could change the taxonomic profile found with 16S sequencing compared to snap frozen samples [35]. However, Anderson et al. observed no difference between baseline and snap frozen or between baseline and stabilized samples with OMNIGENE gut collecting device [16]. Finally, Lin et al. [17] found that samples stabilized with PERFORMABIOME gut were similar to baseline samples after 60 days or after 14 days and 6 freeze-thaw cyles. However, unstabilized samples were different from baseline, snap frozen and stabilized samples, as Lin et al. also found that faeces samples left unstabilized for 14 days had a shift of their microbial community and Roesch et al. [36] found similar results with samples left unstabilized for hours.

Bacterial functions found in the microbiome were also affected by DNA stabilization as shown in Fig. 4. Similar effects were observed by Jenkins et al. [37] who noticed that certain metabolic pathways, inferred via 16S DNA sequencing, were differentially found between stabilized and unstabilized samples. Ma et al. have shown that some bacteria implicated in production of short chain fatty acids (SCFAs) and bile acid metabolism were detected in higher quantity in stabilized samples [38]. Our study, using whole genome sequencing, was not only able to confirm that functions were impacted, but also identified specific functions that were causing the variations between samples, as shown in Table 2. Among other things, an enrichment of some functions related to mobile DNA elements when samples are not stabilized is observed. In addition, the SseB protein is also significantly enriched in unstabilized samples. The fact that this protein is exclusively found in *Enterobacteriaceae* suggests that these microorganisms could be artificially over-represented when the samples are not stabilized. *Enterobacteriaceae* are known to be reservoirs of antibiotic resistance genes in the microbiota of pigs [39,40] and humans [41,42] and their monitoring is crucial for controlling antibiotic resistance. This statement is moreover compatible with the enrichment in mobile elements. However, further studies will be necessary to determine more precisely the impact of stabilization on *Enterobacteriaceae*.

## Conclusion

Stabilization of samples with PERFORMAbiome gut collecting tubes facilitates extraction of higher quality DNA and preservation of the microbial community and its functions. Stabilized samples seem more resistant to freezing/thawing cycles as well. It is a viable option to stabilize previously frozen samples during thawing. This will protect the samples during the following procedures and from future freeze-thaw cycles, which improves DNA preservation.

## Ethics statement

Samples analysed were a subset from a larger pig experiment where the animals were treated as required by the guidelines of the National Farm Animal Care Council (CCAC, 2009). The experimental design was also approved by the Animal Protection Committee of Université Laval prior to the experiments (2019057-1).

## Data availability statement

The whole genome shotgun sequencing data set was deposited in the NCBI's Sequence Read Archive (SRA) database under the accession ID PRJNA801645.

## CRediT authorship contribution statement

**Xavier C. Monger:** Conceptualization, Data curation, Writing – original draft, Writing – review & editing. **Linda Saucier:** Conceptualization, Data curation, Writing – original draft, Writing – review & editing, Supervision, Funding acquisition. **Alex-An Gilbert:** Conceptualization, Data curation, Writing – original draft, Writing – review & editing. **Antony T. Vincent:** Conceptualization, Data curation, Writing – original draft, Writing – review & editing, Supervision, Funding acquisition.

## Declaration of Competing Interests

The authors declare that they have no known competing financial interests or personal relationships that could have appeared to influence the work reported in this paper.
